# Research on Data Fusion Scheme for Wireless Sensor Networks with Combined Improved LEACH and Compressed Sensing

**DOI:** 10.3390/s19214704

**Published:** 2019-10-29

**Authors:** Yu Song, Zhigui Liu, Xiaoli He, Hong Jiang

**Affiliations:** 1 School of Information Engineering, South West University of Science and Technology, Mianyang 621010, China; hexiaoli@suse.edu.cn (X.H.); jianghong@swust.edu.cn (H.J.); 2 Department of Network Information Management Center, Sichuan University of Science and Engineering, Zigong 643000, China; 3 Artificial Intelligence Key Laboratory of Sichuan Province, Sichuan University of Science and Engineering, Zigong 643000, China; 4 School of Computer Science, Sichuan University of Science and Engineering, Zigong 643000, China

**Keywords:** wireless sensor networks, data fusion, clustering routing, compressive sensing

## Abstract

There are a lot of redundant data in wireless sensor networks (WSNs). If these redundant data are processed and transmitted, the node energy consumption will be too fast and will affect the overall lifetime of the network. Data fusion technology compresses the sampled data to eliminate redundancy, which can effectively reduce the amount of data sent by the node and prolong the lifetime of the network. Due to the dynamic nature of WSNs, traditional data fusion techniques still have many problems. Compressed sensing (CS) theory has introduced new ideas to solve these problems for WSNs. Therefore, in this study we analyze the data fusion scheme and propose an algorithm that combines improved clustered (ICL) algorithm low energy adaptive clustering hierarchy (LEACH) and CS (ICL-LEACH-CS). First, we consider the factors of residual energy, distance, and compression ratio and use the improved clustered LEACH algorithm (ICL-LEACH) to elect the cluster head (CH) nodes. Second, the CH uses a Gaussian random observation matrix to perform linear compressed projection (LCP) on the cluster common (CM) node signal and compresses the N-dimensional signal into M-dimensional information. Then, the CH node compresses the data by using a CS algorithm to obtain a measured value and sends the measured value to the sink node. Finally, the sink node reconstructs the signal using a convex optimization method and uses a least squares algorithm to fuse the signal. The signal reconstruction optimization problem is modeled as an equivalent $\ell_{1}$-norm problem. The simulation results show that, compared with other data fusion algorithms, the ICL-LEACH-CS algorithm effectively reduces the node’s transmission while balancing the load between the nodes.

## 1. Introduction

A wireless sensor network (WSN) is a multi-hop self-organizing network system consisting of a large number of sensor nodes with sensing, computing, and communication functions [1]. This type of network is widely used in environmental monitoring, agricultural production, defense military surveillance, industrial automation control, medical monitoring, intelligent robots, and smart cities (such as intelligent transportation, smart homes, and smart travel). Nodes typically have low power and battery-powered features, and once the battery is exhausted, it is unrealistic to replace or charge the battery. Therefore, effectively reducing the energy consumption of WSNs remains a focus of current research [2].

WSNs process specific information in the network coverage area through cooperative sensing, information collection, and data fusion (aggregation) and transmit the information to the base station (BS) by multi-hop forwarding [3]. Data fusion is used to process redundant data or information to combine more energy-efficient and more accurate information in order to improve the efficiency of data collection. Data fusion plays an important role in the research of WSNs and is an effective method to reduce the energy consumption of WSNs [4].

Data fusion can effectively decrease the amount of data transmission and reduce energy consumption. However, due to the strong dynamics of WSNs, solving the problem of the real-time accuracy and reliability of data fusion has become a research hotspot. Compressed (or compressive) sensing (CS) as a new research direction of information science has provided new ideas for solving these problems [5,6]. Therefore, how to apply the theory of compressed sensing to WSN data fusion has become an important research topic.

### 1.1. Related Works

At present, there are many algorithms for WSN data fusion. These studies focus on the following aspects: energy-balanced data fusion [7,8], tree-based data fusion [9], performance-based data fusion [10,11], and security-based data fusion [12,13,14]. These data fusion solutions have driven the development of WSNs. However, the data fusion efficiencies of these traditional data fusion algorithms were generally not high. At the same time, the above studies were based on the Nyquist sampling theorem, which meant that the original signal could be reconstructed from the sampled signal only when the sampling frequency was higher than twice the maximum frequency of the original signal [5]. Of course, this theorem is still valid. However, in many new fields, this method will greatly increase the cost and waste of studies and may even fail to reach the sampling frequency due to hardware limitations. Scientists such as E. J. Candes, J. Romberg, T. Tao, and D. L. Donoho proposed the CS theory in 2004, which can acquire discrete samples of the signal at a much smaller rate than the Nyquist sampling rate, ensuring distortion-free reconstruction of the signal [6]. As a new sampling theory, CS has always been the focus of academia and industry [15,16]. Therefore, CS is considered when studying the data fusion of WSNs to reduce the amount of data transmission.

Recently, the application of CS theory in the data fusion of WSNs has been studied [17,18]. The research in reference [17] studied the problem of joint sparse support recovery with 1-bit quantized compressive measurements in a distributed sensor network. For sparsity pattern recovery with 1-bit quantized measurements, the study of reference [18] have developed two computationally tractable centralized algorithms (i.e., MLA and C-BIHT) and proposed a new iterative dictionary learning algorithm. It can be used to reconstruction the noise sparse signal under a one-bit CS framework without previously unknown the sparse domain. Using the dictionary learning algorithm to train the two matrices (i.e., the measurement matrix and sparse domain matrix), the experimental results showed that the algorithm was effective for a case with no dictionary learning. 

The above methods gradually add the information of each node to the data being transmitted by encoding during the process of data transmission, thereby solving the problem of an unbalanced network load. However, due to the large number of nodes and the wide distribution in the WSNs, the node can potentially transmit the gathered data directly to the sink node without increasing the energy consumption; this is an urgent problem that needs to be solved. Data fusion based on clustering routing has been proposed and has received extensive attention. In the study of [19], Heinezlinan proposed a low energy adaptive clustering hierarchy (LEACH) algorithm, which is the first hierarchical routing protocol for data fusion and is a classic clustering protocol. It solves the problem of the limited network scale caused by the planar routing protocol and supports larger scale networks. 

Energy is limited in the WSNs, so how to choose an effective data fusion solution is especially important. The idea of combining clustering algorithm and CS technology to process the information has become a new trend in WSNs data fusion research [20,21,22,23,24,25,26]. The research in reference [20] investigated an energy-efficient data aggregation scheme to reduce communication costs and prolong the network’s lifetime for a cluster-based underwater acoustic sensor network inspired by the theory of distributed compressed sensing (DCS). A joint reconstruction model highly consistent with JSM-2 and the observation matrix were newly designed in [20]. The study in reference [21] introduced clustering-based CS on the basis of clustering (cluster compression sensing, CCS) to further compress the data. Since the CH of CCS sent the linear compressed projection information to the sink node, the linear compressed projection projected the high-dimensional signal to the low-dimensional measurement value, and the information reconstruction of the sink node increased the dimension from low-dimensional to high-dimensional.

The authors of reference [22] proposed a novel energy-efficient CS-based clustering routing (EECSR) protocol that combined the merits of the clustering strategy and the CS-based scheme. To alleviate the “hot spot problem” and reduce the energy consumption resulted from the rotation of the role of CHs, a third role of backup CH (BCH) as well as the corresponding mechanism to rotate the roles between the CH and BCH was proposed in reference [22]. Experiments shown that the EECSR was superior to the existing clustering algorithms and the CS-based algorithms in terms of energy efficiency and extending the lifetime of WSNs. The study in reference [23] investigated a kind of effective data aggregating method based on the CS and the clustering. The aggregation process was divided into two parts, i.e., intra-cluster and inter-cluster. In the cluster, the sink node sent the seed vector to each CH. The CH generated the corresponding random interval sparse matrices and collected data by CH. Among clusters, the cluster nodes forwarded the measurements along the multi-hop routing tree to the sink node. In this paper, we divide the data fusion process into two parts (i.e., in the cluster and among the clusters) according to the idea of reference [22]. The study in reference [24] focused on the energy-efficient data gathering method based on the integration of the clustering and the CS. In a clustered WSN, the CH collected the data from the non-CH nodes, added all received data to its own data, and then sent the combined measurements to the BS. Experimental verification was performed using K-means and LEACH. However, the energy consumption of multi-hop inter-cluster communication in the network is not considered in reference [24].

The most relevant research to our work is reference [25]. A routing algorithm, based on a dual CH redundant mechanism combined with a CS data fusion algorithm, was proposed in reference [25]. A dual CH alternation mechanism was used to balance the energy consumption of the CH node. Eliminating redundancy through CS data fusion technology effectively improved the reliability of WSNs and reduced data redundancy. Inspired by reference [25], we combine clustering routing, data fusion, and CS. The CH selection strategy of reference [25] was similar to a traditional WSN clustering routing scheme, but the difference was that a dual CH alternation mechanism was considered. Although this dual CH mechanism could alleviate the burden on the CH node, it created additional energy consumption. Therefore, designing an effective data fusion scheme based on clustering routing and CS while considering node energy efficiency is an important area of research for WSNs. To the best of our knowledge, this work remains an open topic for those who study WSN data fusion. 

### 1.2. Contributions

In this research, we investigate a clustering data fusion scheme based on CS for WSNs. We aim to reduce the node energy consumption, reduce the amount of data transmission, improve the signal reconstruction accuracy, and prolong the network’s lifetime. Thus, the main innovations of this paper can be summarized as follows:

- Using the spatial correlation between nodes in WSNs, this paper proposes a clustering data fusion method based on an improved LEACH clustering protocol and sparse hybrid CS. The data fusion process is divided into two parts, clustering and CS.
- In the clustering process, this paper improves the shortcomings (e.g., clustering is not uniform, and it is easy to make a node repeat when the CH energy’s premature consumption is completed as a dead point) of the LEACH algorithm. We consider the influence of residual energy, distance, and compression ratios on the CH node selection and propose a new threshold function.
- In the CS process, we convert the solution of the $\ell_{0}$-norm to the solution of the $\ell_{1}$-norm and transform the non-convex optimization problem into a convex optimization problem. The convex optimization Lagrangian dual function is used to solve the optimization problem and reconstruct the sensor node information. This algorithm has a fast convergence speed and low time complexity. At the same time, it is possible to optimize the network topology and balance the energy consumption of each node in the network.
- The performance analysis and comparison of the experimental results and related methods show that the proposed algorithm can reduce the sampling information of the nodes and better reconstruct the node source, thereby making the network more adaptable and robust.

### 1.3. Organization

The remainder of this paper is organized as follows. In Section 2, the system model is introduced. The algorithm (i.e., the clustering algorithm and compressive sensing data fusion algorithm) for a WSN scenario is designed and implemented in Section 3. Numerical results are presented in Section 4. Finally, Section 5 concludes the paper.

## 2. System Model

### 2.1. Network Model

**Figure 1:**
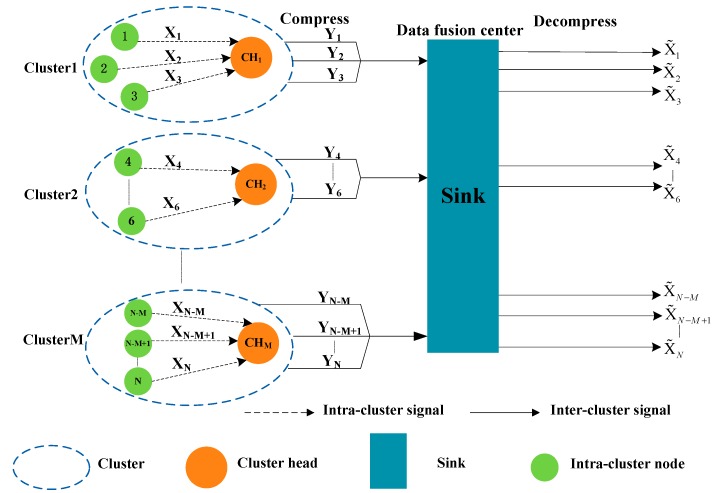
Wireless sensor network (WSN) system model based on clustering data fusion with compressed sensing (CS).

In this paper, we consider a distributed WSN scenario, in which energy is limited. Therefore, choosing an effective energy-saving clustering algorithm and data fusion algorithm is very important for solving WSN data transmission and data processing. The system model diagram is shown as Figure 1. More specifically, we assume that there are a total of N sensor nodes in the network, which are randomly and evenly deployed within the region, and the sink node is at the center.

We can see from Figure 1 that, with the clustering algorithm, the nodes of the WSNs are divided into M clusters, and each cluster has a CH node, where $\Phi_{CH}=\left\{1,2,\ldots ,M\right\}$ is a set of CH. In the cluster, the i−th node transfers the data $X_{i}\left(0\leq i\leq N\right)$ to the CH node in a single hop manner. The CH node observes the gathered information $Y_{i}\left(0\leq i\leq N\right)$ and then transfers the observations to the aggregation node (i.e., the sink node), which recovers the original data estimated value ${\;\tilde{X}}_{i}\left(0\leq i\leq N\right)$ via the reconstruction algorithm. At the same time, we also assume that the nodes in each cluster are evenly distributed—that is, there is one CH node in each cluster, and there are n=NM−1 cluster member (CM) nodes. In this paper, it is assumed that there are two levels of transmissions, namely, intra-cluster (i.e., nodes to CH node) signal transmission without CS, and inter-cluster (i.e., CH node to sink node) signal transmissions based on CS. Data are transmitted in a single hop, either within the cluster or outside the cluster.

### 2.2. Energy Consumption Model

In this paper, energy consumption includes the required energy for transmitting, receiving, and processing data packets. According to the classical wireless communication energy consumption model [19], we assume that the energy of the transmitter includes both signal processing and power amplification, while the energy of the receiver is only used for signal processing. The Euclidean theory can be used to describe the distance between two nodes. Then, the distance between node i and node j can be expressed as follows:

(1) $$d_{ij}=\sqrt{{\left(d_{i}-d_{j}\right)}^{2}+{\left({\hat{d}}_{i}-{\hat{d}}_{j}\right)}^{2}},\forall i,j\in \left\{1,2,\cdots ,N\right\}$$

where the node i and node j can also be denoted as ($d_{i},{\hat{d}}_{i}$) and ($d_{j},{\hat{d}}_{j}$).

We assume that the free space model is used when the distance between the CM nodes and the CH node is less than $d_{0}$, and the multipath fading model is used when the distance between the CH node and the sink node is greater than $d_{0}$. 

The energy that consumed by the transmission of l-bit messages between the two nodes with a distance of d is as follows:

(2) $$\begin{array}{ll} E_{TX} & =\underset{\mathrm{energy}\;\mathrm{consumed}\;\mathrm{by}\;\mathrm{transmitting}\;\mathrm{circuit}}{\underbrace{E_{TX-elec}}}+\underset{\mathrm{energy}\;\mathrm{consumed}\;\mathrm{by}\;\mathrm{amplifying}\;\mathrm{circuit}\;\mathrm{loss}}{\underbrace{E_{TX-amp}}}\\ & =\left\{ \begin{array}{l} lE_{elec}+l\varepsilon_{fs}d^{2},d<d_{0},\;\mathrm{intra}-\mathrm{cluster}\;(\mathrm{CM}\;\mathrm{to}\;\mathrm{CH})\\ lE_{elec}+l\varepsilon_{mp}d^{4},d\geq d_{0},\;\mathrm{inter}-\mathrm{cluster}\;(\mathrm{CH}\;\mathrm{to}\;\mathrm{Sin}k) \end{array}\right. \end{array}$$

where d0=εfsεmp, $E_{elec}$ is the energy required for signal processing, and the amplifier power consumption $\varepsilon_{fs}$ and $\varepsilon_{mp}$ are determined by the transmission distance and the received bit error rate.

The energy consumed by the l-bit messages’ reception is as follows:

(3) $$E_{RX}=\underset{\mathrm{energy}\;\mathrm{consumed}\;\mathrm{by}\;\mathrm{reception}\;\mathrm{circuit}}{\underbrace{E_{RX-elec}}}=lE_{elec}$$

When a message is transmitted by means of cluster communication, the total energy consumption of the network is composed of the energy consumption of CH and CM. Furthermore, the energy consumption of the intra-cluster CH node consists of receiving the data from the CM node, and data fusion. While the energy consumption of the inter-cluster CH node consists of the transmitting data to the sink and the CS. The specific energy consumption is as follows:

(4) $$\begin{array}{ll} E_{CH_{i}-intra} & =\underset{\mathrm{energy}\;\mathrm{consumed}\;\mathrm{by}\;\mathrm{reception}}{\underbrace{E_{CH_{i}-RX-in\begin{array}{c} tra \end{array}}}}+\underset{\mathrm{energy}\;\mathrm{consumed}\;\mathrm{by}\;\mathrm{data}\;\mathrm{fusion}}{\underbrace{E_{CH_{i}-DF-in\begin{array}{c} tra \end{array}}}}\\ & =nlE_{elec}+\left(n+1\right)lE_{df}\\ & =l\left(nE_{elec}+\left(n+1\right)E_{df}\right) \end{array}$$

where n is the number of the CM nodes, and $E_{df}$ is the data fusion cost of 1-bit messages in a cluster. 

Likewise, the energy consumption of the inter-cluster CH node is given by Equation (5).

(5) $$\begin{array}{ll} E_{CH_{i}-inter} & =+\underset{\mathrm{energy}\;\mathrm{consumed}\;\mathrm{by}\;\mathrm{transmitting}}{\underbrace{E_{CH_{i}-TX-inter}}}+\underset{\mathrm{energy}\;\mathrm{consumed}\;\mathrm{by}\;\mathrm{compressed}\;\mathrm{sensing}}{\underbrace{E_{CH_{i}-CS-inter}}}\\ & =\left(n+1\right)\alpha \left(lE_{elec}+l\varepsilon_{mp}d_{CH_{i}to\mathrm{si}nk}^{4}\right)+\left(n+1\right)lE_{cs}\\ & =\left(n+1\right)l\left(\alpha \left(E_{elec}+\varepsilon_{mp}d_{CH_{i}to\mathrm{si}nk}^{4}\right)+E_{cs}\right) \end{array}$$

where α is the compressed ratio, which is related to CS, $d_{CH_{i}to\mathrm{si}\begin{array}{c} nk \end{array}}$ is the distance between the i-th CH and the sink, and $E_{cs}$ is the compressed sensing cost of 1-bit messages in a cluster.

Hence, according to Equations (4) and (5), the total energy consumption of the i-th CH will be as follows:

(6) $$\begin{array}{ll} E_{CH_{i}} & =E_{CH_{i}-intra}+E_{CH_{i}-inter}\\ & =l\left(nE_{elec}+\left(n+1\right)E_{df}\right)+\left(n+1\right)l\left(\alpha \left(E_{elec}+\varepsilon_{mp}d_{CH_{i}to\mathrm{si}nk}^{4}\right)+E_{cs}\right)\\ & =l\left(\left(nE_{elec}+\left(n+1\right)E_{df}\right)+\left(n+1\right)\left(\alpha \left(E_{elec}+\varepsilon_{mp}d_{CH_{i}to\mathrm{si}nk}^{4}\right)+E_{cs}\right)\right) \end{array}$$

Meanwhile, the total energy consumption of the CM nodes in the i-th cluster is calculated by Equation (7):

(7) $$E_{CM-CH_{i}}=\sum_{j=1}^{n}E_{CM_{j}-CH_{i}}=\sum_{j=1}^{n}l\left(E_{elec}+\varepsilon_{fs}d_{CM_{j}toCH_{i}}^{2}\right)$$

where $E_{CM_{j}-CH_{i}}$ is the amount of energy consumed by the j-th CM node in the i-th cluster, and $d_{CM_{j}toCH_{i}}$ is the distance between the j-th CM and the i-th CH.

As a result, the total energy consumption of the WSNs is expressed according to in Equation (8).

(8) $$\begin{array}{ll} E_{total} & =\sum_{i=1}^{M}\left(E_{CH_{i}}+E_{CM-CH_{i}}\right)\\ & =\sum_{i=1}^{M}\left(l\left(\left(nE_{elec}+\left(n+1\right)E_{df}\right)+\left(n+1\right)\left(\alpha \left(E_{elec}+\varepsilon_{mp}d_{CH_{i}to\mathrm{si}nk}^{4}\right)+E_{cs}\right)\right)+\sum_{j=1}^{n}l\left(E_{elec}+\varepsilon_{fs}d_{CM_{j}toCH_{i}}^{2}\right)\right)\\ & =l\sum_{i=1}^{M}\left(\left(\left(nE_{elec}+\left(n+1\right)E_{df}\right)+\left(n+1\right)\left(\alpha \left(E_{elec}+\varepsilon_{mp}d_{CH_{i}to\mathrm{si}nk}^{4}\right)+E_{cs}\right)\right)+\sum_{j=1}^{n}\left(E_{elec}+\varepsilon_{fs}d_{CM_{j}toCH_{i}}^{2}\right)\right) \end{array}$$

Then, we can obtain the residual energy of the CH node and CM node in the r-th round, as follows:

(9) $$\begin{array}{c} E_{CH_{i}-residual}^{r}=\left\{ \begin{array}{l} \min\left(E_{\max},E_{CH_{i}-residual}^{r-1}-E_{CH_{i}}^{r}\right),r>0\\ E_{\mathrm{initial}}\;,r=0 \end{array}\right. \end{array}$$

(10) $$\begin{array}{c} E_{CM-CH_{i}-residual}^{r}=\left\{ \begin{array}{l} \min\left(E_{\max},E_{CM-CH_{i}-residual}^{r-1}-E_{CM-CH_{i}}^{r}\right),r>0\\ {\hat{E}}_{\mathrm{initial}}\;,r=0 \end{array}\right. \end{array}$$

where $E_{\mathrm{initial}}$ and ${\hat{E}}_{\mathrm{initial}}$ are the initial energies of the CH and CM nodes, respectively; $E_{\max}$ is the maximum capacity of the battery; and $E_{CH_{i}}^{r}$ and $E_{CM-CH_{i}}^{r}$ are the energy consumption of the CH and CM nodes in the r-th round.

### 2.3. CS Data Fusion Model

**Figure 2:**
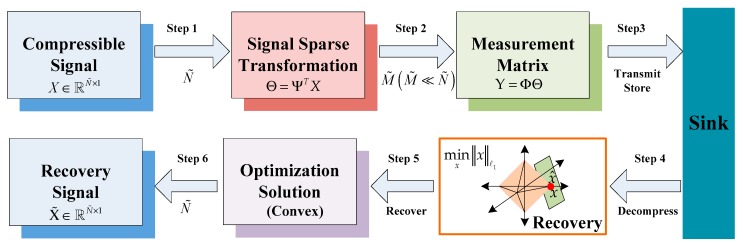
Compressed sensing data fusion model.

In this section, we will introduce CS theory and its application in WSNs. The model diagram combining CS theory and WSN data fusion is shown in Figure 2.

As can be seen from Figure 2, the idea of CS data fusion theory is as follows. For signal $X\in {\mathbb{R}}^{\tilde{N}\times 1}$, we will first judge whether it is sparse or compressible. If it is not a sparse signal, then we need to find a sparse basis $\Psi \in {\mathbb{R}}^{\tilde{N}\times \tilde{N}}$, which makes X sparse basedon the orthogonal transform basis Ψ. The sparse signal Θ (i.e., Θ is K sparse.) and the measurement matrix $\Phi \in {\mathbb{R}}^{\tilde{M}\times \tilde{N}}(\tilde{M}\ll \tilde{N})$ are operated to obtain a measurement value $Y\in {\mathbb{R}}^{\tilde{M}\times 1}$, provided that the measurement matrix Φ is not related to the sparse base Ψ (i.e., the restricted isometry property (RIP) is satisfied). The sink node performs data fusion on the received signals. It receives and stores the low-dimensional measurement vector Y of the original signal X and recovers the original signal using a recovery algorithm. We intend to use the convex algorithm to optimize the solution and recover the original signal to get $\tilde{X}$.

As can be seen from Figure 2, there are three important aspects in the research of CS data fusion—signal sparse transformation (the orthogonal basis for sparse representation), measurement matrix design (the measurement matrix satisfying the RIP property) [6], and signal reconstruction (a robust and efficient reconstruction algorithm). Next, we will introduce these three aspects in detail.

#### 2.3.1. Signal Sparse Transformation

Suppose any compressible signal $X={\left[x_{1},x_{2},\cdots ,x_{\tilde{N}}\right]}^{T}$ of length $\tilde{N}$ can be represented by Equation (11).

(11) $$X=\sum_{i=1}^{\tilde{N}}\psi_{i}\theta_{i}\mathrm{or}\;X=\Psi \Theta$$

(12) $$\theta_{i}=\langle X,\psi_{i}\rangle \mathrm{or}\;\Theta =\Psi^{T}X$$

where X and Θ are different expressions of the signal X in two different transform domains (i.e., the time domain and the frequency domain). $\Psi =\left[\psi_{1},\psi_{2},\cdots ,\psi_{\tilde{N}}\right]$ is a set with a standard orthogonal basis. $\psi_{i}$ is the column of Ψ. $\Theta ={\left\{\theta_{i}\right\}}_{i=1}^{\tilde{N}}$ is the column vector of element *θ*_*i*_. If most of the elements of the vector Θ are 0, then X is called sparse. The number of non-zero is denoted as $K\left(K\approx \tilde{M}\ll \tilde{N}\right)$, i.e., ${\left\|\Theta \right\|}_{0}=K$, and X is said to be K sparse.

#### 2.3.2. Measurement Matrix Design

If the projection vector Θ is strictly sparse or compressible, then we can perform a compression measurement. Compression measurement is a process of linearly measuring a high-dimensional original signal to obtain a low-dimensional vector. The matrix $\Phi ={\left[\phi_{1},\phi_{2},\cdots \phi_{\tilde{M}}\right]}^{T}$ in Figure 2 is the measurement matrix. The restricted isometry property (RIP) [6] theory has proven that the necessary and sufficient condition for accurately recovering signal X is that Φ and Ψ are irrelevant. In this case, we can project the original signal X via the measurement matrix Φ to obtain the measurement vector $Y=\left[y_{1},y_{2},\cdots y_{\tilde{M}}\right]$. The inner product $y_{j}$ of X and ${\left\{\phi_{j}\right\}}_{j=1}^{\tilde{M}}$ can then be obtained from Equation (13).

(13) $$y_{j}=\langle X,\phi_{j}\rangle \left(j=1,2,\cdots \tilde{M}\right)$$

According to Equations (11)–(13), the following expression can be obtained:

(14) $$Y=\Phi X=\Phi \Psi \Theta =\hat{\Phi }\Theta$$

where Y is the column vector of $\tilde{M}\times 1$, Φ is the measurement matrix of $\tilde{M}\times \tilde{N}\left(\tilde{M}\ll \tilde{N}\right)$, and $\hat{\Phi }=\Phi \Psi \in {\mathbb{R}}^{\tilde{M}\times \tilde{N}}$ is the “sensing” matrix. M˜N˜ is called the compression ratio, which reflects the compression performance of the CS algorithm. The lower the compression ratio, the lower the cost of sampling the original signal, and the more efficient the compression. 

#### 2.3.3. Signal Reconstruction

In this section, the signal reconstruction (i.e., we will recover $X\in {\mathbb{R}}^{\tilde{N}\times 1}$ from $Y\in {\mathbb{R}}^{\tilde{M}\times 1}\left(\tilde{M}\ll \tilde{N}\right)$) remains a serious problem. Since the original signal X is K sparse in a certain transform domain, Equation (14) has a unique sparse solution when $K\approx \tilde{M}\ll \tilde{N}$. Therefore, when the “sensing” matrix $\hat{\Phi }\in {\mathbb{R}}^{\tilde{M}\times \tilde{N}}$ satisfies the RIP constraints (Please refer to Appendix A for the details on RIP), the under-determined equation problem of the Equation (14) can be transformed into the minimum $\ell_{0}$-norm problem.

(15) $$\begin{array}{l} \min{\left\|\Psi^{T}X\right\|}_{\ell_{0}}\\ s.t.Y=\hat{\Phi }\Theta \end{array}$$

The $\ell_{0}$-norm represents the number of non-zero entries in the vector, which is a non-deterministic polynomial (NP) hard problem. Therefore, the solution to the $\ell_{0}$-norm can be equivalent to the solution of the $\ell_{1}$-norm, that is, the non-convex optimization problem is transformed into a convex optimization problem. Consequently, we can recover X from Y by solving the following convex optimization algorithm:

(16) $$\begin{array}{l} \min{\left\|\Psi^{T}X\right\|}_{\ell_{1}}\\ s.t.Y=\hat{\Phi }\Theta \end{array}$$

## 3. Algorithm Design and Implementation

The energy of the nodes in WSNs is limited. Designing an efficient energy-saving clustering algorithm and data fusion algorithm will greatly reduce the energy consumption of the network. Therefore, in this section, we introduce the clustering algorithm and the clustering data fusion algorithm based on CS. The detailed algorithm is as follows. The network model is shown in Figure 1. After choosing the CH nodes and the forming clusters, all nodes in each cluster send packets to the CH node in a single hop manner. Then, the CH node sends the fused packets to the sink node by single hop or multi-hop. Finally, the sink node chooses a reasonable CS reconstruction algorithm to reconstruct the network data according to the characteristics of the network and the node data.

### 3.1. Improved LEACH Cluster Algorithm

The LEACH algorithm is an adaptive clustering topology algorithm. The idea of the LEACH algorithm is to randomly select the CH node in a round manner and distribute the energy load of the entire network evenly to each sensor node in the network, thereby reducing the network’s energy consumption and improving the network’s lifetime. Compared with other planar multi-hop routing protocols and static layering algorithms, LEACH could extend the network’s lifetime by 15%. Although LEACH can improve the lifetime of WSNs, there are still some problems, as follows.

- In the LEACH algorithm, each round of loops must reconstruct the cluster, and the energy cost of constructing the cluster is relatively large.
- Since the LEACH algorithm assumes that all nodes can communicate directly with the sink node, this protocol is not suitable for use in large-scale WSNs.
- The LEACH algorithm does not consider the current energy status of the CH node. If the node with lower energy is selected as the CH node, it might accelerate the death of the node and affect the lifetime of the entire network.
- The LEACH algorithm does not consider the distance between the CH node and the sink node. When the CH node is far away from the sink node, it still uses single-hop communication, which causes the node to consume significant energy and even exhausted energy.
- The number and distribution of the CH nodes were not considered. Therefore, there might be an unbalanced distribution of the selected CH nodes. In some places, there were many CH nodes, and some places did not have any CH nodes.

Considering the above deficiencies of the LEACH algorithm, in this paper, the chance that each node is selected as CH is determined according to the residual energy of the node and the communication distance. A node that can be selected as a CH node with a high probability has a minimum time delay compared to its neighbor nodes. Based on the LEACH protocol, this paper proposes an improvement clustering algorithm (ICL-LEACH) that comprehensively considers the residual energy, communication distance, and data fusion rate of nodes. The energy of this algorithm is sufficient, its energy consumption is slow, the threshold for a node to have a good position and high data fusion rate is larger, and its chance of being the CH is increased, so that the network load is evenly distributed and the network’s lifetime is prolonged. 

#### 3.1.1. Setup Phase

At the initial phase of the CH election, the node i randomly picks a number between 0 to 1. If the number is less than the threshold values $T_{i}$, then node i becomes the CH in this current round. Considering the influence of the residual energy, distance and compression ratio on the selection of CH nodes, this paper introduces an improved threshold function $T_{i-new}$, which is defined as in Equation (17).

(17) $$\begin{array}{c} T_{i-new}=\left\{ \begin{array}{l} \frac{p}{1-p\times \left(r\mathrm{mod}p^{-1}\right)}\times \omega ,i\in I\\ 0,otherwise \end{array}\right. \end{array}$$

where p is the expected ratio that selected as a CH node, and r is the current round number. $\left(r\mathrm{mod}p^{-1}\right)$ is the number of the CH in this CH selection period. I is the set of nodes that have not been selected as the CH node in the last *p*^−1^ round, and ω is the value of a dynamic variable, which is defined as in Equation (18),

(18) $$\begin{array}{c} \omega =\lambda_{1}\times \frac{E_{i-residual}}{E_{i-\max}} \end{array}+\lambda_{2}\times \frac{d_{\max}-d_{i-to\sin k}}{d_{\max}}+\lambda_{3}\times \alpha$$

(19) $$\alpha =\frac{l_{original}}{l_{current}}$$

(20) $$\begin{array}{c} \lambda_{1} \end{array}+\lambda_{2}+\lambda_{3}=1$$

where $E_{i-residual}$ and $E_{i-\max}$ are the residual energy and the maximum energy of node i, respectively. This may increase the probability that a node with higher residual energy is selected as a CH. $d_{i-to\sin k}$ and $d_{\max}$ are the distance from node i to the sink node and the maximum distance from the entire node to the sink node, respectively. When the distance from the node to the sink node is too large, it needs to consume too much energy, and the probability that this node is selected as the CH is also greatly reduced. $l_{current}$-bits and $l_{original}$-bits are the current and original transmission message lengths of the node. α is the compression ratio. When α is higher, the probability that the node is selected as the CH is also higher. $\lambda_{1}$, $\lambda_{2}$, and $\lambda_{3}$ are the weighting factors that represent the relationship between the energy, distance, and compression.

Once the CH node is selected, the CH broadcasts the selected message to all the nodes. The other common node selects the cluster to be joined according to the strength of the received signal and sends the information to the corresponding CH node. Then, the CH node uses time division multiple access (TDMA) to allocate the time slots for transmitting data to the member nodes in the cluster.

#### 3.1.2. Stabilization Phase

In the stabilization phase, the member nodes in the cluster transfer the collected data to the CH node. When the CH node receives the data sent by all the nodes in the cluster, the CH node performs information fusion on the data. The fused data are then transmitted to the sink node. The sink node transmits the data to the monitoring center for data processing. Since the direct data transmission of each cluster is not performed separately, the communication in each cluster may be affected by the adjacent cluster. Therefore, each cluster communicates with different code division multiple access (CDMA) codes to reduce the interference of other cluster nodes. After the stabilization phase continues for a period of time, the network re-enters the cluster setup phase and begins to select a new round of CH nodes, which continually circulate. In order to save the resource overhead, the duration of the stabilization phase is longer than the duration of the setup phase during each round of clustering. 

**Figure 3:**
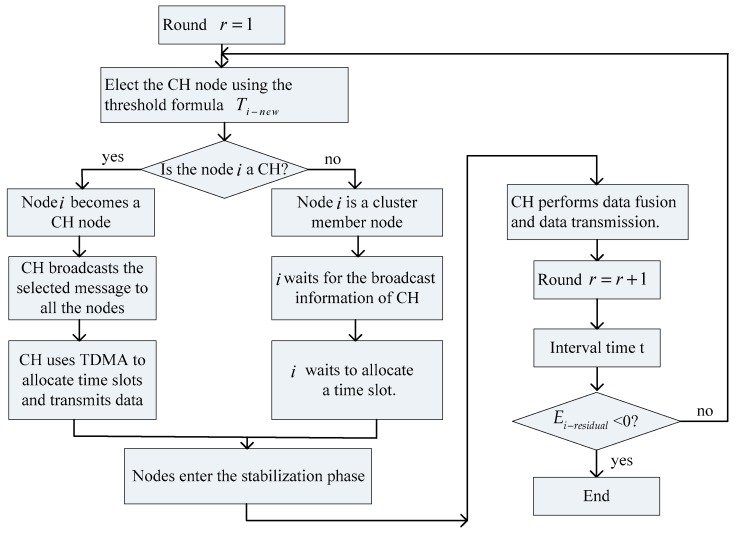
The improved clustered low energy adaptive clustering hierarchy(ICL-LEACH) algorithm flow chart.

In general, the process of the ICL-LEACH algorithm can be described as the process in Figure 3, which clearly illustrates the concept of the algorithm.

### 3.2. Clustering Data Fusion Algorithm Based on Compressive Sensing

In WSNs, the energy resources are limited and valuable. Thus, the use of energy-saving algorithms (i.e., the combination of the clustering algorithm and the CS algorithm) to process the information can effectively reduce the network’s energy consumption. In this section, we will use CS for data fusion after the clustering in the previous section. For the physical characteristics of the WSNs, the CH node completes the data compression processing, and the sink node completes the data reconstruction.

#### 3.2.1. Selection of the Sparse Transform Basis

According to the system model diagram of Figure 1, it can be seen that, with the ICL-LEACH algorithm, the WSN is divided into M clusters. In other words, there are M CH nodes. We use $CH_{m}(m\in \Phi_{CH})$ to indicate the m−th CH node. In the m-th cluster, the $CH_{m}$ node collects the data in its own cluster, and the $i-\mathrm{th}\left(0\leq i\leq n=\tilde{N}\right)$ node (i.e., $CM_{i}$) transfers the sensing signal $x_{mi}$ to the $CH_{m}$ node. At time t, the signal sent by each node to the $CH_{m}$ in the cluster is $X_{m}={\left[x_{m1},x_{m2},\cdots ,x_{m\tilde{N}}\right]}^{T}$. $X_{m}\in {\mathbb{R}}^{\tilde{N}\times 1}$ is a time-dependent $\tilde{N}$-dimensional discrete signal. We assume that, in this node-intensive WSN, the signals perceived by the nodes have a spatial-temporal correlation [26]. Generally speaking, signals are not sparse in the time domain. Therefore, before applying CS theory, the signal must first be sparsely transformed to find its sparse basis. Typical signals are sparse on a Wavelet transform basis, a fast Fourier transform basis, and a discrete cosine transform basis. In this paper, we choose the classical Fourier transform sparse method. This method has the advantages of good stability and low computational complexity. Signal sparse representation is as follows:

(21) $$X_{m}=\Psi_{m}\Theta_{m}=\left[\psi_{m1},\psi_{m2},\cdots ,\psi_{m\tilde{N}}\right]\left[\begin{array}{l} \theta_{m1}\\ \theta_{m2}\\ \cdots \\ \theta_{m\tilde{N}} \end{array}\right]=\sum_{i=1}^{\tilde{N}}\psi_{mi}\theta_{mi}$$

where $\Psi_{m}\in {\mathbb{R}}^{\tilde{N}\times \tilde{N}}$ is the standard orthogonal basis in the m−th cluster, and if $X_{m}$ is sparse, then $\Psi_{m}$ is the identity matrix. $\Theta_{m}\in {\mathbb{R}}^{\tilde{N}\times 1}$ represents the coefficient of the signal $X_{m}$ on this sparse basis, and $\Theta_{m}$ has $K\left(K\approx \tilde{M}\ll \tilde{N}\right)$ non-zero elements. $\Theta_{m}$ can also be expressed in Equation (22).

(22) $$\Theta_{m}=\Psi_{m}{}^{T}X_{m}$$

#### 3.2.2. Measurement Matrix Optimization

If the signal $X_{m}\in {\mathbb{R}}^{\tilde{N}\times 1}$ can be represented by a sparse basis $\Psi_{m}\in {\mathbb{R}}^{\tilde{N}\times \tilde{N}}$, then $X_{m}$ can be compressed. Specifically, we can project signal $X_{m}$ to get the main data without losing important information. Therefore, it is very important to design a suitable measurement matrix. The measurement matrix not only reduces the number of dimensions but also minimizes the loss of information of the original signal $X_{m}$. We generate a Gaussian random measurement matrix $\Phi_{m}\in {\mathbb{R}}^{\tilde{M}\times \tilde{N}}$ at the $CH_{m}$ and use $\Phi_{m}\in {\mathbb{R}}^{\tilde{M}\times \tilde{N}}$ to project $X_{m}\in {\mathbb{R}}^{\tilde{N}\times 1}$ to obtain $Y_{m}\in {\mathbb{R}}^{\tilde{M}\times 1}$.

(23) $$Y_{m}=\Phi_{m}X_{m}+N_{m}=\Phi_{m}\Psi_{m}\Theta_{m}+N_{m}={\hat{\Phi }}_{m}\Theta_{m}+N_{m}$$

where $N_{m}$ denotes a noise vector which will be the zero vector when we regard the noiseless case. The component of $N_{m}$ obeys a Gaussian independent distribution of random variables of $N\left(0,\sigma^{2}\right)$, and ${\hat{\Phi }}_{m}\in {\mathbb{R}}^{\tilde{M}\times \tilde{N}}=\Phi_{m}\Psi_{m}$ is the “sensing” matrix. We can write Equation (23) in the form of Equation (24), as below.

(24) $$\underset{Y\text{:}\tilde{M}\times 1}{\underbrace{\left[\begin{array}{l} y_{m1}\\ y_{m2}\\ \cdots \\ y_{m\tilde{M}} \end{array}\right]}}=\underset{\Phi :\tilde{M}\times \tilde{N}}{\underbrace{\left[\begin{array}{l} \phi_{11}\phi_{11}\cdots \phi_{1\tilde{N}}\\ \phi_{21}\phi_{22}\cdots \phi_{2\tilde{N}}\\ \phi_{\tilde{M}1}\phi_{\tilde{M}2}\cdots \phi_{\tilde{M}\tilde{N}} \end{array}\right]}}\underset{\tilde{N}\times 1}{\underbrace{\left[\begin{array}{l} x_{m1}\\ x_{m2}\\ \cdots \\ x_{m\tilde{N}} \end{array}\right]}}+\underset{\tilde{M}\times 1}{\underbrace{\left[\begin{array}{l} \sigma_{1}\\ \sigma_{2}\\ \cdots \\ \sigma_{\tilde{M}} \end{array}\right]}}=\underset{\Phi :\tilde{M}\times \tilde{N}}{\underbrace{\left[\begin{array}{l} \phi_{11}\phi_{11}\cdots \phi_{1\tilde{N}}\\ \phi_{21}\phi_{22}\cdots \phi_{2\tilde{N}}\\ \phi_{\tilde{M}1}\phi_{\tilde{M}2}\cdots \phi_{\tilde{M}\tilde{N}} \end{array}\right]}}\underset{\Psi :\tilde{N}\times \tilde{N}}{\underbrace{\left[\begin{array}{l} \psi_{m1}\begin{array}{c} 0 \end{array}\cdots 0\\ 0\psi_{m2}\cdots 0\\ 00\cdots 0\\ 00\cdots \psi_{m\tilde{N}} \end{array}\right]}}\underset{\Theta :\tilde{N}\times 1}{\underbrace{\left[\begin{array}{l} \theta_{m1}\\ \theta_{m2}\\ \cdots \\ \theta_{m\tilde{N}} \end{array}\right]}}+\underset{\tilde{M}\times 1}{\underbrace{\left[\begin{array}{l} \sigma_{1}\\ \sigma_{2}\\ \cdots \\ \sigma_{\tilde{M}} \end{array}\right]}}$$

It can be seen from Equations (15) and (24) that if the K sparse signal $X_{m}$ is to be accurately reconstructed to obtain ${\tilde{X}}_{m}$, the number of measurements $\tilde{M}$ must satisfy the following conditions [6]:

(25) M˜=O(Klog(N˜K))

#### 3.2.3. Reconstruction Algorithm

The reconstruction algorithm is the core content of CS. In this section, we will use the solution of the $\ell_{1}$-norm problem to accurately reconstruct signal $X_{m}$ to get ${\tilde{X}}_{m}^{*}$.

(26) $${\tilde{X}}_{m}^{*}=\arg\min{\left\|X_{m}\right\|}_{\ell_{0}}s.t.Y_{m}=X_{m}\Theta_{m}$$

The minimum $\ell_{1}$-norm problem is a convex optimal problem. By applying the Lagrange multiplier, the optimization problem can be transformed into an unconstrained convex optimization problem,

(27) $$L(X_{m})={\left\|X_{m}\right\|}_{\ell_{1}}^{2}+\lambda_{m}\left(X_{m}\Theta_{m}-Y_{m}\right)$$

where $\lambda_{m}$ is the Lagrange multiplier, satisfying $\lambda_{m}\geq 0$ at the same time. By using the Lagrange dual constraint decomposition method, a sub-gradient iterative algorithm is constructed to solve the dual problem. The dual function of Equation (27) is shown as

(28) $$D(\lambda_{m})=\min L\left(X_{m}\right)$$

The dual problem of Equation (28) can be written as follows:

(29) $$\begin{array}{l} \max D(\lambda_{m})\\ s.t.\lambda_{m}\geq 0 \end{array}$$

In order to obtain the optimal value ${\tilde{X}}_{m}^{*}$, $X_{m}$ can be derived according to the Karush-Kuhn-Tucker (KKT) condition with a value of zero.

(30) $$\frac{\partial L\left(X_{m}\right)}{\partial X_{m}}=2X_{m}+\Theta_{m}{}^{T}\lambda_{m}=0$$

Then, we get the optimal solution

(31) $${\tilde{X}}_{m}^{*}=-\frac{1}{2}\Theta_{m}{}^{T}\lambda_{m}$$

Here, it is worth noting that for the optimal value ${\tilde{X}}_{m}^{*}$, we use the reconstruction algorithm to get signal ${\tilde{X}}_{m}$. In practical applications, there is a certain error between the original signal and the reconstructed signal. Therefore, the quality of the reconstruction algorithm determines the size of the error. To verify the performance of our proposed reconstruction method, we use the following two evaluation criterions in this paper: the signal-to-noise ratio (*SNR*) and root mean-squared error (*RMSE*).

(32) $$SNR=10\times \mathrm{lg}\left(\frac{{\left\|X_{m}\right\|}_{2}}{{\left\|X_{m}-{\tilde{X}}_{m}\right\|}_{2}}\right)$$

(33) $$RMSE=\frac{1}{\sqrt{l}}\sum_{i=1}^{l}{\left\|X_{mi}-{\tilde{X}}_{mi}\right\|}_{2}$$

where $X_{m}$ is the original sensor data of the m−th cluster, ${\tilde{X}}_{m}$ represents the reconstructed value of the m−th cluster, ${\left\|\cdot \right\|}_{2}$ denotes the minimization, l is the length of the data signal, and $X_{mi}$ and ${\tilde{X}}_{mi}$ are the original data signal and the reconstructed value of the i−th node in the m−th cluster, respectively. It can be concluded from Equation (32) that the bigger the *SNR* is, the better performance the algorithm will be.

#### 3.2.4. Algorithm Process

Our proposed algorithm consists of three sections—(1) CH node election, (2) CH node data compression, and (3) sink node reconstruction data. In this way, the algorithm processes for the clustering data fusion algorithm based on CS are described in Algorithm 1, Algorithm 2, and Algorithm 3, respectively. The detailed pseudo-code for the three algorithms are as follows.

### 3.3. Algorithm Analysis

#### 3.3.1. Algorithm Convergence Analysis

The algorithm convergence and the convergence speed are important indicators for evaluating the performance of the algorithm. The algorithm convergences fast, which not only shortens the CH election time, but also improves data fusion efficiency. In order to analyze the convergence, we will discuss the convergence of the ICL-LEACH algorithm and the CS data fusion algorithm, respectively.

**Algorithm 1** ICL-LEACH Algorithm for CH Node Election **1:Require:**N, M, n, r, R, p, G, $T_{i-new}$, $d_{\max}$, $d_{ij}$, $\lambda_{1}$, $\lambda_{2}$, $\lambda_{3}$, $E_{i-residual}$, $E_{i-\max}$, $rand_{i}$, $type_{i}=no$, $CH_{i}=no$, $l_{original}$, $l_{current}$ **2:Ensure:**M **3:While**1≤r≤R and $E_{i-residual}>0$
**do** **4:**
**If**
$CH_{i}=no$ **5:**  **For**
i=1 to N do **6:**  **If**
$rand_{i}\leq T_{i-new}$ **7:**
$type_{i}=CH$ and $CH_{i}=yes$ **8:**  CHnodebroadcasts the selected message to all the nodes **9:**  CH node uses TDMA to allocate time slots and transmits data **10:**   **Else**
$type_{i}=CM$ and $CH_{i}=no$ **11:**  CM node waits for the broadcast information of CH node **12:**  CM node waits to allocate a time slot **13:**   **End if** **14:**    nodes enter the stabilization phase **15:**  **End for** **16:  Else** CH node performs data fusion and data transmission **17:**
**End if** **18:**
r←r+1 **19:Endwhile**

**Algorithm2** CH Node Data Compressed Algorithm**1: Require:**$X_{m}\in {\mathbb{R}}^{\tilde{N}\times 1}$, $x_{mi}$, $Y_{m}\in {\mathbb{R}}^{\tilde{M}\times 1}$, $y_{mj}$, $\Phi_{m}\in {\mathbb{R}}^{\tilde{M}\times \tilde{N}}$, $\phi_{mji}$, $\sigma_{mj}$, $\Theta_{m}\in {\mathbb{R}}^{\tilde{N}\times 1}$, $\theta_{mi}$, $\Psi_{m}\in {\mathbb{R}}^{\tilde{N}\times \tilde{N}}$, $\psi_{mii}$ **2: Ensure:**$Y_{m}$ **3:**  **While**
1≤m≤M
**do** **4:**    **For**
j=1 to $\tilde{M}$ do **5:**     **For**
i=1 to $\tilde{N}$ do **4:**      $X_{m}=\sum_{i=1}^{\tilde{N}}\psi_{mii}\theta_{mi}$ **6:**     **End for** **7:**  $y_{mj}=\langle X_{m},\phi_{mji}\rangle +\sigma_{mj}$ **8:**    **End for** **9:**   CH node sends $Y_{m}$ to the sink node **10:**
**Endwhile**

**Algorithm 3** Sink Node Reconstruction Signal**1: Require:**$X_{m}$, $x_{mi}$, $Y_{m}$, $y_{mj}$, $\Phi_{m}$, $\phi_{mji}$, $\sigma_{mj}$, $\Theta_{m}$, $\Psi_{m}$, $\psi_{mii}$, ξ, ${\hat{\Phi }}_{m}$, **2: Ensure:**${\tilde{X}}_{m}$ **3: When** the sink node received $Y_{m}$ from the m−th CH node **then** **4:**  Reconstruct the signal using the CVX tool to solve the $\ell_{1}$-norm minimum **5:**  **Cvx_begin** **6:**   variable $X_{m}$ **7:**   minimize (norm ($\Psi_{m}^{T}X_{m}$,1)) **8:**   subject to **9:**   $Y_{m}={\hat{\Phi }}_{m}\Theta_{m}+\sigma_{m}$ **10:**  **Cvx_end** **11:**  **IF**
$\left|X_{m}-{\tilde{X}}_{m}\right|>\xi$
**then** **12:**   change the CH nodes based on the new source **13:   Else** go to the Algorithm 1 **14:**  **End if** **15:**
**End**

As described above, the key points of our proposed algorithm include clustering, sparse coding, dictionary learning, and reconstruction. Therefore, the discussion of the convergence of the proposed algorithm is actually a discussion of the convergence of the improved clustering algorithm and the CS algorithm. Firstly, the ICL-LEACH algorithm is based on LEACH. The convergence of the LEACH algorithm has been proven in many well-known papers [19,27]. Therefore, the ICL-LEACH algorithm is convergent. Secondly, in the iterative scheme of convex optimization, the convergence speed depends on the distance from the starting point to the optimal solution [25]. To speed up convergence, we take advantage of the overlap between windows and use the starting point. By selecting the starting point, the expected number of iterations of convergence can be reduced, resulting in a good estimate of $X_{m}$. We use this formula to prove the convergence of the CS data fusion algorithm. See Appendix B for specific certification procedures. A comprehensive analysis can guarantee the convergence of our proposed algorithm. 

#### 3.3.2. Algorithm Time Complexity Analysis

Time complexity is another indicator used to evaluate the efficiency of an algorithm. According to Algorithm 1, Algorithm 2, and Algorithm 3, the time complexity of our proposed algorithm also includes three parts: the clustering algorithm, compression algorithm and reconstruction algorithm. After analysis, the time complexity of the proposed algorithm is as follows.

The time complexity of the clustering process is O(R×N), the time complexity of the compression algorithm is $O\left(M\times \left(\tilde{N}+\tilde{M}\right)\right)$, and the time complexity of the reconstruction algorithm is $O\left(\tilde{M}\right)$, where R is the largest round of clustering, N is the number of sensor nodes, *M* is the number of clusters, $\tilde{N}$ is the dimension, and $\tilde{M}$ is the dimension. Then, it can be seen that the overall time complexity of the algorithm is $O\left(R\times N\right)+O\left(M\times \left(\tilde{N}+\tilde{M}\right)\right)+O\left(\tilde{M}\right)$.

## 4. System Simulation Analysis

In this section, we use a numerical simulation to evaluate the performance of our proposed algorithm ICL-LEACH-CS. As shown in Figure 1, it is assumed that the coverage of the WSN is 100 (m) × 100 (m). Further 100 wireless sensor nodes are randomly distributed in this area, and a sink node is located at the center of this area. The simulation parameters used in this paper (according to [9,20,21,27,28]) are shown in Table 1. In order to verify the performance of the algorithm, two experimental methods (i.e., experimental environment establishment and MATLAB simulation) are adopted. The $\ell_{1}$-Magic toolbox and convex optimization toolbox of MATLAB 2015b are used to solve the $\ell_{1}$-norm optimization [29].

### 4.1. Experimental Setup

#### 4.1.1. Experimental Environment

**Figure 4:**
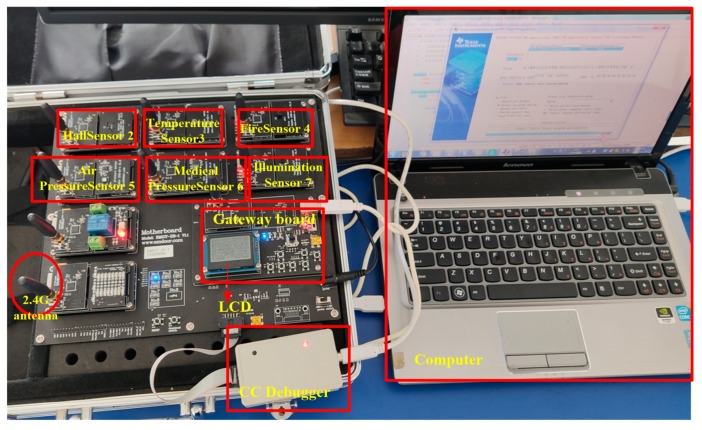
Building an experimental platform.

To better verify the performance of our proposed algorithm, we built an experimental environment to test whether it could be deployed in a true WSN. We built the experimental environment with the EMIOT-JX-1, which integrates multiple sensor models and multiple wireless networking modes to run multiple internet of things (IoT) network architectures. The experimental environment is shown in the Figure 4. The specific steps to set up the experimental environment are as follows:

- Install the CC-Debug emulator driver;
- Install the CP210X_vcp_win_xp driver for COM 3;
- Install the CC-Debug emulator driver;
- Install the CP210X_vcp_win_xp driver, the simulation is serial port 3;
- Install Setup_SmartRFProgr;
- Install monitoring software ZigbemPC;
- Install the TinyOS development environment program ZigbemDS.

Without loss of generality, in this part of the experiment we chose six wireless sensor nodes (i.e., Hall Sensor, Medical Pressure Sensor, Air Pressure Sensor, Fire Sensor, Temperature Sensor, Illumination Sensor) for research. The results of this study can also be extended to the scenarios of multiple wireless sensor nodes. The WSN experiment requires the construction of the TinyOS development environment. TinyOS is an open source IoT operating system that supports CC2430 chip, supports ultra-low power RF transmission, supports mobile independent network, and supports Zigbee protocol. The main development tool is ZigbemDS, which installation package automatically installs rich components into the TinyOS development environment for developers to use.

#### 4.1.2. Experimental Process

**Figure 5:**
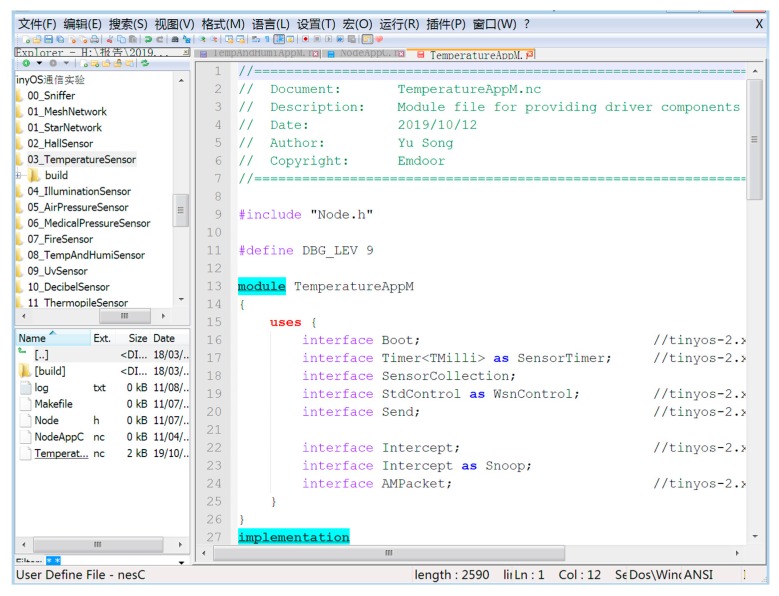
Compile the program.

We use a CC2430 module to burn the program into the gateway board node and the six sensor nodes, respectively. As shown in Figure 5, we open the code compiler Notepad++ for ZigbemDS (DonHO, Taiwan, China) to compile our program.

**Figure 6:**
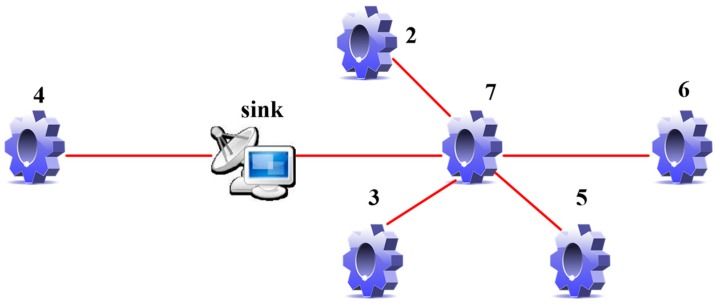
A topology of six wireless sensor network (WSN) sensor nodes.

Open the ZigbemPC program to observe the operation of the WSN. ZigbemPC is EMIOT-JX-1 PC terminal control software, which is mainly used for map monitoring, display of sensor instant data, and display of network topology. As shown in Figure 6, a six-node WSN topology can be seen in the ZigbemPC program. Node 4 (i.e., FireSensor) is directly connected to the sink node, and node 7 (i.e., IlluminationSensor), node 2 (i.e., HallSensor), node 3 (i.e., TemperatureSensor), node 5 (i.e., Air PressureSensor, FireSensor), and node 6 (i.e., Medical PressureSensor) form a cluster. Node 7 is a CH node and is directly connected to the sink node. The other four nodes send information to node 7. 

**Figure 7:**
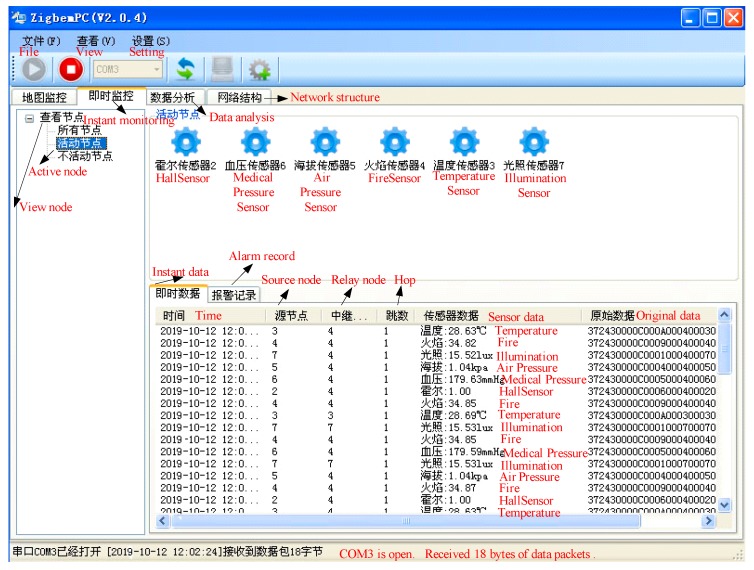
ZigbemPC monitoring data.

As shown in Figure 7, we can get the change of data packets between the six nodes by observing ZigbemPC. At the same time, the source node, the relay node, the number of hops the data of different sensor nodes, and the original data can all be seen. Instant data is comprehensive and can be analyzed with our results.

**Figure 8:**
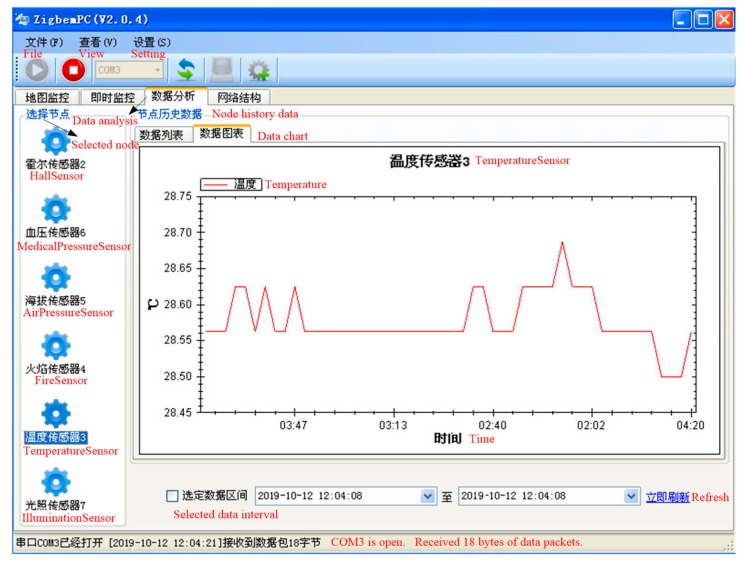
The data chart for node 3.

Taking node 3 (i.e., Temperature Sensor) as an example, we perform data analysis on it. Through ZigbemPC, we can see the temperature change in node 3 during the observation time. The data chart of node 3 is shown in Figure 8. We burn the algorithm into node 3, and node 3 can run normally, which can prove the correctness of our algorithm.

Through the compilation and operation of the program in the COM debugging assistant, we can see the basic functions of the ICL-LEACH-CS algorithm, i.e., clustering and transmission. The program is then burned into the sensor node. We use the monitoring software ZigbemPC to see how the program is operating in the sensor node. There are many packets between the sensor nodes, and the size of the packets sent from the source to the CH node is normal. The experimental results verify the correctness of our proposed algorithm ICL-LEACH-CS.

In order to evaluate the performance of the ICL-LEACH-CS algorithm, we will carry out simulation analysis in two parts—a clustering algorithm performance analysis and a CS data fusion performance analysis. The specific performance analysis is as follows. 

### 4.2. Performance Evaluation

#### 4.2.1. ICEL-LEACH-CS Algorithm Clustering Process

The simulation scenarios in this paper are set as follows:

- The sensor nodes are randomly distributed in a square area of 100 (m) × 100 (m);
- WSN is a homogeneous network with each sensor node having the same function and a unique number;
- The sensor node energy is limited, and it does not have an energy harvesting function;
- After the sensor node is deployed, its location is fixed;
- The sink node is located in the center of the area, i.e., the coordinates of the sink node are (50, 50), and the location is also fixed;
- The node communicates with the CH node through a single hop, and the CH node communicates with the sink node in a single hop or multi-hop manner.

**Figure 9:**
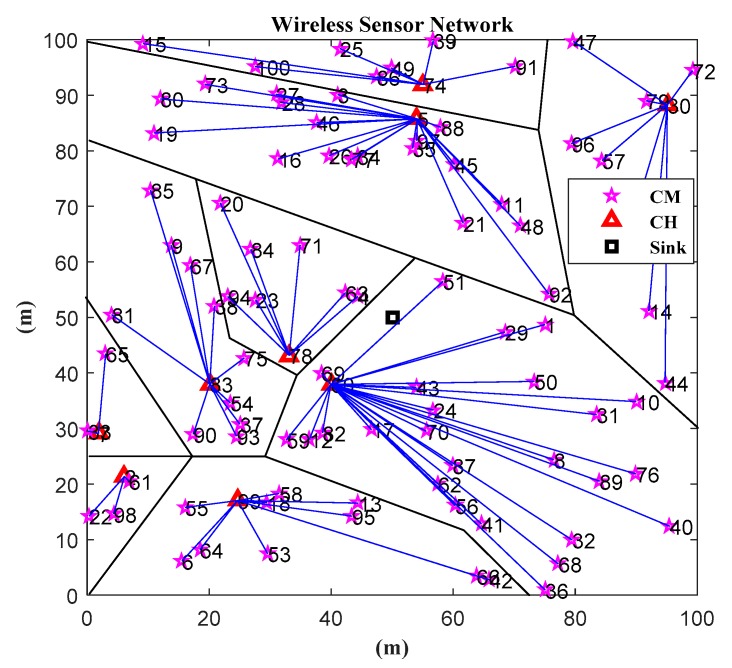
WSN topology and ICL-LEACH-CS algorithm clustering process.

Specific parameters are shown in Table 1. As can be seen from Figure 9, the distribution of the nodes is not very uniform. At the same time, it also can be seen that, by running the ICL-LEACH-CS algorithm, the 100 wireless sensor nodes are divided into nine clusters, including nine CH nodes. Each CM node directly communicates with the CH node, and the CH node communicates with the sink node through the CS data fusion. In this way, redundant data transmission can be reduced, the residual energy of the nodes can be saved, and the lifetime of the WSN network can be improved [30].

#### 4.2.2. Comparison with Five-Type Cluster Strategy

In order to better and more accurately evaluate the advantages and disadvantages of our proposed ICL-LEACH-CS algorithm, we compare it with LEACH [27], LEACH-C [19], DHLCS [25], LEACH-FL [31], and LEACH-GA [32] from the WSN’s lifetime and residual energy. Among them, LEACH-C is an improved algorithm for LEACH. LEACH-C extends the random CH selection algorithm of LEACH via deterministic components. The authors of reference [25] proposed a WSN routing protocol (DHLCS) based on an alternate dual CH mechanism and compressive sensing. LEACH-FL is an improved algorithm of LEACH using fuzzy logic (FL) [31]. The mechanism is similar to LCH, but the variables (parameters) used are power, distance, and node density. Experiments show that the LEACH-FL algorithm has a lower energy consumption rate and longer network’s lifetime than the LEACH algorithm. LEACH-GA is another improved algorithm of LEACH using genetic algorithm (GA) [32]. In each round, the LEACH-GA has three phases—set-up, steady-state, and preparation. The preparation phase is only performed once before the first round of set-up phase. The set-up and steady-state process for each round is the same as LEACH.

**Figure 10:**
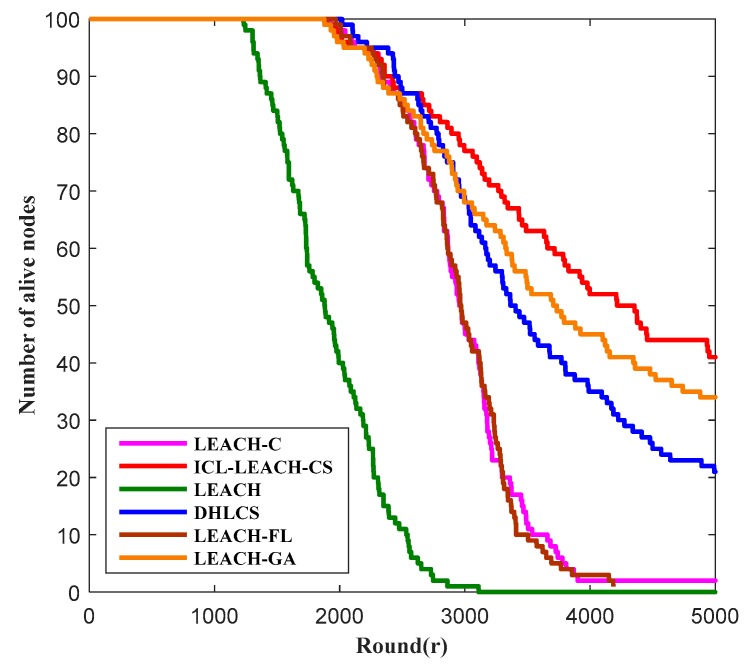
Number of alive nodes vs. round.

As shown in Figure 10, we compare ICL-LEACH-CS with five other algorithms (i.e., LEACH-C, LEACH-CS, DHLCS, LEACH-FL, and LEACH-GA) to see how the number of surviving sensor nodes in the WSN varies with the number of rounds. As can be seen from Figure 10, the maximum number of rounds is 5000, and the number of network nodes is 100. When the number of rounds is about 1250, the number of surviving nodes of the six algorithms is 100. After this round, the number of surviving nodes of the LEACH algorithm begins to decline. The LEACH-C algorithm, DHLCS algorithm, ICL-LEACH-CS, LEACH-FL, and LEACH-GA algorithms have little difference in performance. However, after 1900 rounds, the number of surviving nodes of the five algorithms also began to decline. It can be seen that DHLCS can balance CH energy consumption by electing double CH nodes. LEACH-GA uses the GA search node to become the best probability of the CH, and the change is also slow by minimizing the total energy consumption required to complete one round of the sensor field. Since LEACH-C and LEACH-FL do not consider data fusion, the energy consumption is relatively large. Thus, the surviving nodes of DHLCS change slowly. Considering the residual energy, distance, and compression ratio, our algorithm has a slower change than the DHLCS algorithm. When the number of rounds is 4000, there are about 51 surviving nodes in our algorithm. Therefore, our algorithm can extend the lifetime of a WSN.

**Figure 11:**
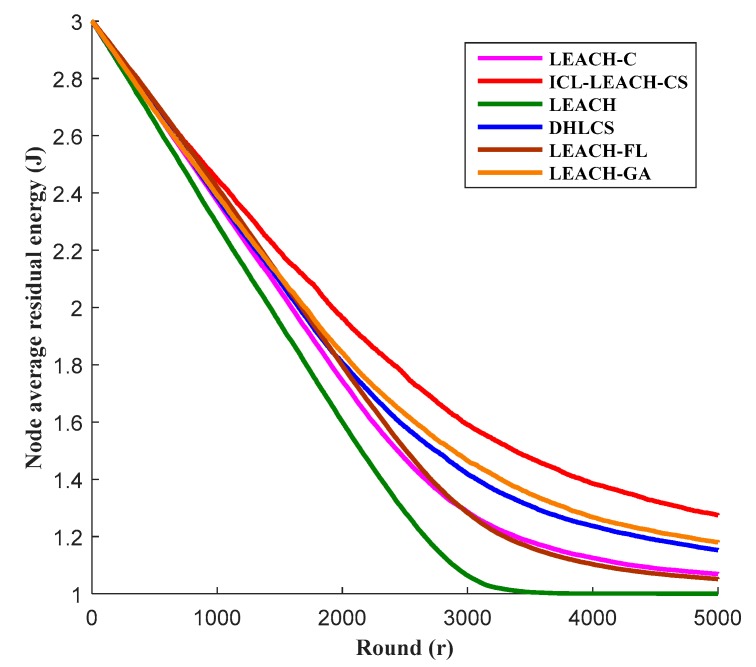
Node average residual energy vs. round.

The election of the CH nodes in the WSN is very important. Too many or too few CH nodes will cause the energy consumption of the nodes to be too fast. Figure 11 represents the average residual energy of the node as a function of the number of rounds. It can be seen from Figure 11 that LEACH has the least residual energy, followed by LEACH-FL and LEACH-C. Our algorithm ICL-LEACH-CS has the most residual energy. The main reason is that the basic idea of LEACH is to select the CH node by random loop, and the number of CH nodes cannot be guaranteed, so the energy consumption of the algorithm is very fast. However, LEACH-FL is a centralized algorithm that does not have data fusion capabilities. Therefore, it has high energy consumption. LEACH-C has made some improvements to the clustering algorithm, which is no longer the primary way to randomly select CH nodes. The LEACH-C protocol uses nodes with energy no lower than the average energy value as candidate nodes. This method can reduce the energy consumed by communication when electing CH nodes, so more residual energy can be used to transmit data. Although LEACH-GA and DHLCS can achieve better clustering performance, the size distribution of each cluster is more uniform, and the network energy consumption is balanced. However, in terms of the number of clusters per round, our algorithm ICL-LEACH-CS is better than LEACH-GA and DHLCS. Moreover, the data transmission rate of LEACH-GA cannot be estimated [30]. And when using multi-hop transmission, DHLCS does not consider the hotspot near the sink, resulting in a decrease in the residual energy of the network. The ICL-LEACH-CS algorithm proposed in this paper selects the CH node based on the residual energy and communication distance of the node. Therefore, the energy consumption of this algorithm is the slowest, and the residual energy of the nodes is greatly improved [30].

#### 4.2.3. ICEL-LEACH-CS Algorithm Reconstruction Process

In order to verify the reconstruction performance of our CS algorithm, we will conduct two sets of simulation experiments. The first set of experiments is proof of the recovery performance of our CS algorithm. The second set of experiments is a comparison between different CS algorithms.

**Figure 12:**
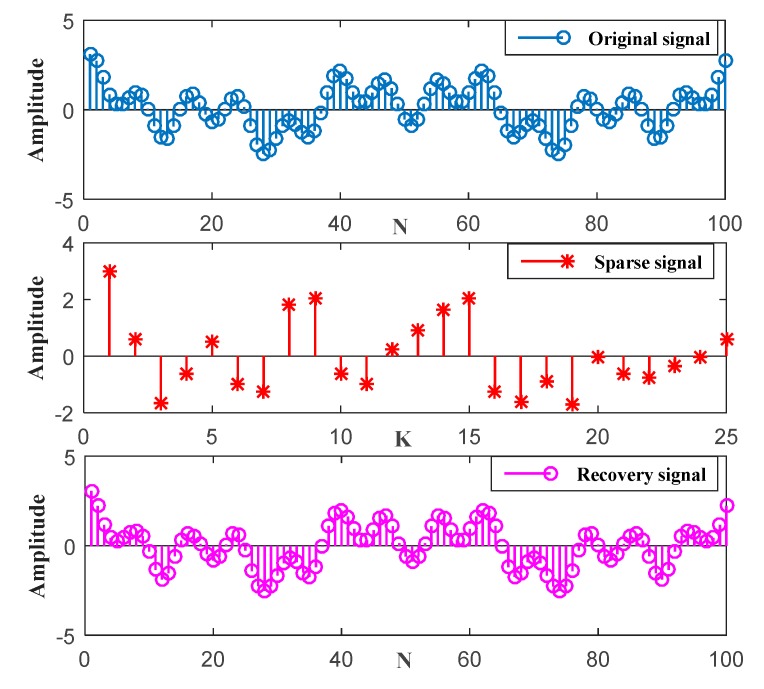
Original signal, sparse signal, and recovery signal.

We consider the one-dimensional signal, as shown in Figure 12, i.e., x(t)=cos(26πt)+cos(10πt)+cos(4πt). In the actual application scenario, the original signal always has noise. As can be seen from Figure 12, we performed CS on the original signal containing noise. First, the original signal is randomly sampled by the sparsity K=25 to obtain discrete samples of the signal. Then, using the $\ell_{1}$, the optimization problem is transformed into convex optimization for reconstruction, and the reconstructed signal is obtained. However, there is an error between the original real signal and the recovery signal because of noise. According to Equation (33), the $RMSE=6.0641\times e^{-14}$ can be obtained and the value of RMSE is very small. Here, we assume that the signal recovery is successful if the RMSE is less than $1\times e^{-9}$. It can be seen that our proposed CS algorithm has strong signal reconstruction capabilities.

**Figure 13:**
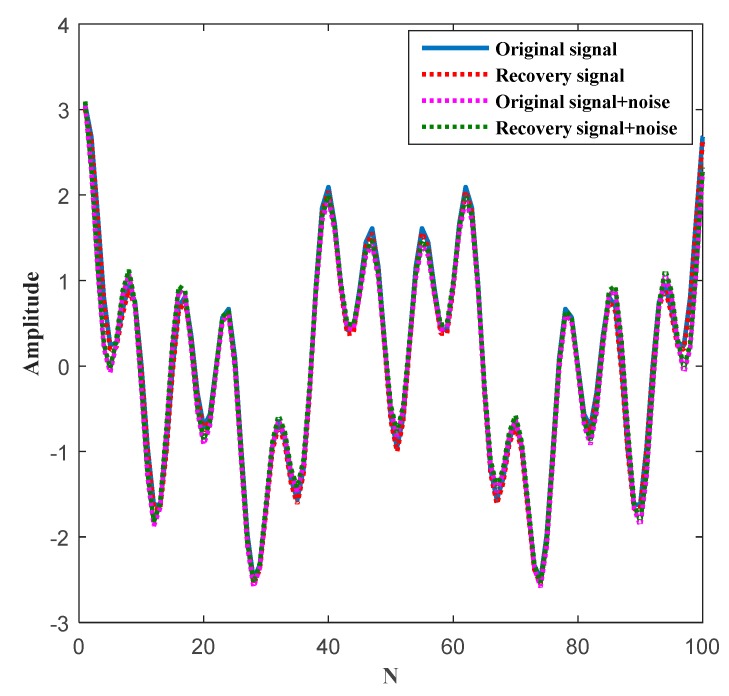
The influence of noise on theoriginal signal and recovery signal.

In general, wireless channel noise is interference for a useful signal. If the noise interference is not removed, the signal will be distorted, and the communication will not be performed correctly and effectively. In order to verify the reconstruction performance of our proposed CS algorithm, we have added the following set of experiments. The original signal is divided into an original signal without noise and an original signal with noise. The recovery signal is divided into a recovery signal without noise and a recovery signal with noise. The noise value is 0.1. As can be seen from Figure 13, at the inflection point of the waveform, the influence of noise on the signal is very obvious. When reconstructing the original signal without noise, the error value is very small, and almost all information can be completely reconstructed. When considering the reconstruction of a noisy original signal, the error value is slightly larger but is within a controllable range. Therefore, our algorithm can efficiently reconstruct the original information in both noisy and noise-free environments. It can effectively reduce the amount of data being transmitted and also improve the accuracy of data transmission.

#### 4.2.4. Comparison with Five-Type Data Fusion Strategy

In WSNs, as network size increases, data fusion and data transmission cannot be ignored. The use of clustering and CS in data fusion can reduce the amount of data being transmitted and balance the traffic load of the entire network. In order to evaluate the performance of our proposed ICL-LEACH-CS algorithm, we intend to compare it with existing data fusion algorithms. These comparative data fusion strategies include the method based on CS and cluster [23], integration of the cluster and CS [24], CS data fusion without cluster [26], cluster data fusion without CS [28], and cluster with hybrid CS [21].

**Figure 14:**
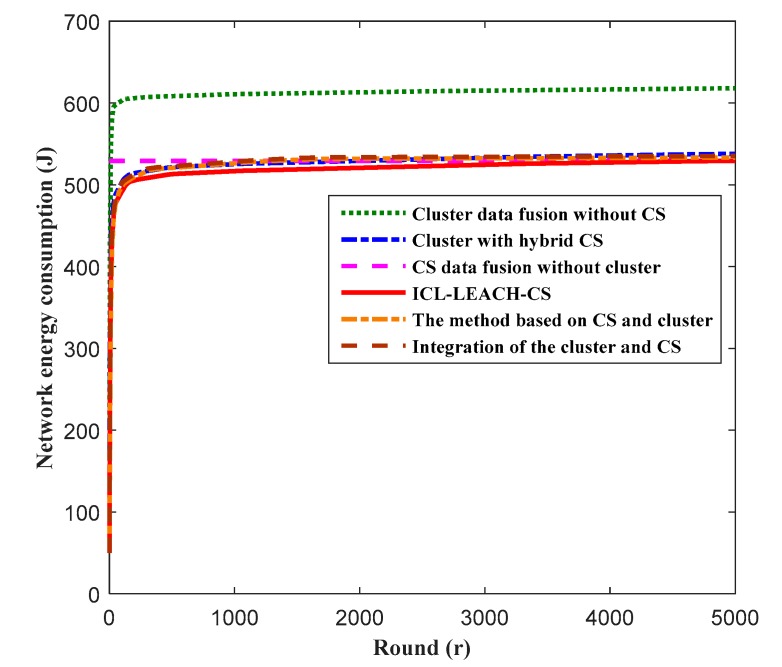
Network energy consumption vs. round.

Figure 14 describes the energy consumption of the network as a function of time. In WSNs, network energy consumption is mainly derived from CM nodes and CH nodes. The energy consumption of the CM node is mainly used for data collection and transmission, and the energy consumption of the CH node is mainly used for data collection, receiving data from the CM node, data fusion processing, and transmitting the fused data to the sink node. Therefore, the data fusion algorithm has an impact on energy consumption. As can be seen from Figure 14, the method based on CS and cluster, integration of the cluster and CS, the cluster with hybrid CS, CS data fusion with cluster, and ICL-LEACH-CS all utilize CS, so the sensor node actually transmits the measured value of the node-aware data. These five algorithms convert high-dimensional data into low-dimensional data, and the transmitted data dimension is much lower than the original node-aware data. Therefore, the energy consumption of the sensor node is much lower than that of the cluster data fusion without CS. Our proposed ICL-LEACH-CS algorithm improves the CH election, so the energy consumption is the lowest. It can be seen that the ICL-LEACH-CS algorithm proposed in this paper is very suitable for a WSN with limited energy.

**Figure 15:**
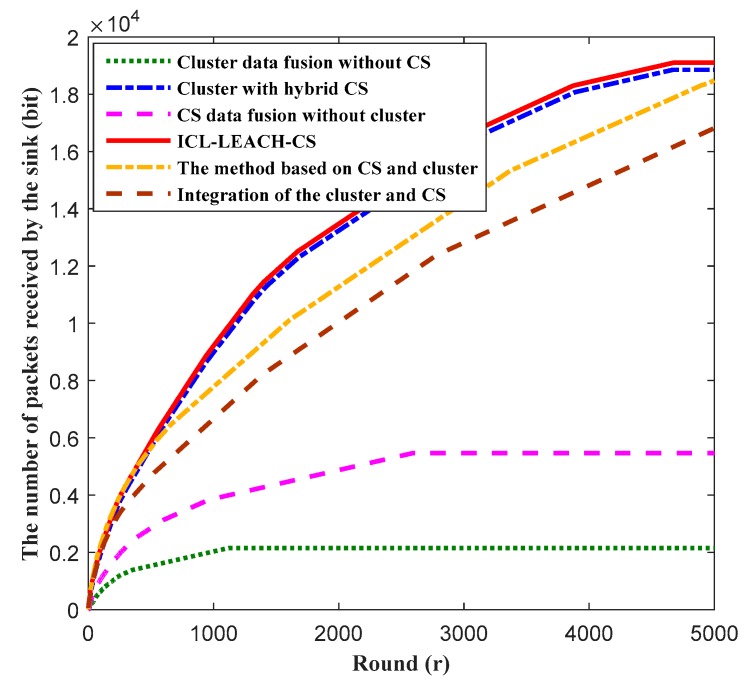
Data transmission at the sink node vs. round.

The lifetime of the WSN affects the amount of data received by the sink node. The longer the lifetime is, the more data the sink node receives. Figure 15 illustrates the number of packets received by the sink nodes of the six algorithms (i.e., the method based on CS and cluster, integration of the cluster and CS, cluster data fusion without CS, cluster with hybrid CS, CS data fusion without cluster, and ICL-LEACH-CS) as the number of rounds increases. It can be seen that, with an increase of rounds, the amount of data received by the sink node also increases. When the round is 2000, our proposed ICL-LEACH-CS algorithm outperforms the other five algorithms (i.e., the method based on CS and cluster, integration of the cluster and CS, cluster data fusion without CS, cluster with hybrid CS, and CS data fusion without cluster). The main reason for this result is that we consider the residual energy and distance. In the CH election, the nodes with lower residual energy are avoided as the CH, so the transmission capacity of the nodes is increased.

**Figure 16:**
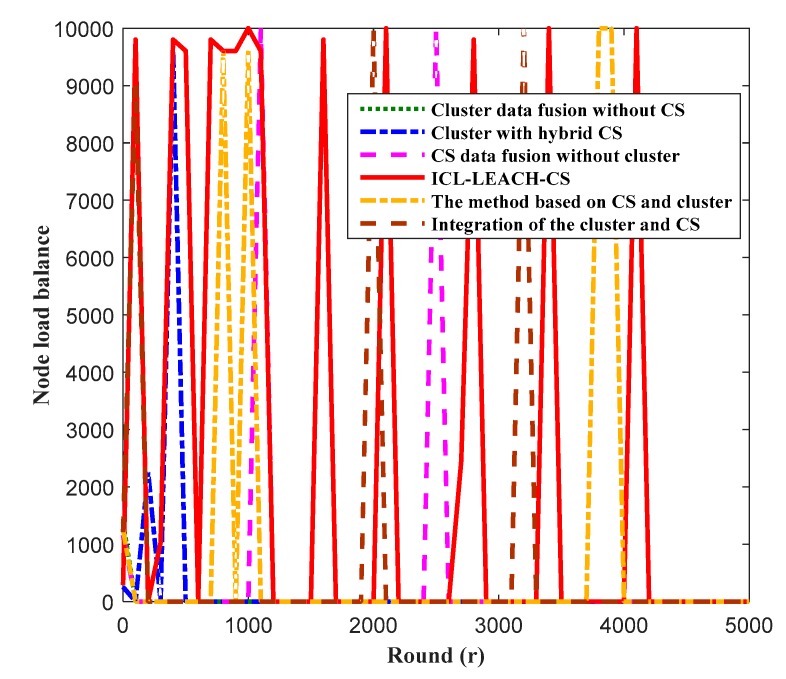
Node load balancevs. round.

In WSNs, the network load balance refers to the balance load (i.e., task or energy consumption) of multiple wireless sensor nodes to coordinate tasks or reduce energy consumption. The clustering algorithm, multi-hop routing algorithm, and data fusion algorithm can solve the problem of the network load balance. However, the traditional clustering algorithm and data fusion algorithm can easily form a “hot zone” phenomenon and an “energy hole” problem. The “hot zone” phenomenon, or “energy hole,” refers to the fact that the node that is closer to the sink node has a larger amount of data to be forwarded, resulting in the energy consumption of the CH node near the sink node being too fast and dying. Figure 16 depicts the load balance change of a node as time increases. In Figure 16, it is clear that the proposed ICL-LEACH-CS load balance is better. This occurs because we have improved the clustering algorithm, considering the distance, residual energy and compression ratio of the nodes. When the CH is elected, avoiding being close to the sink node is always elected as the CH. Multi-hop routing can be performed between the CH nodes, and the distant CH transmits data to the CH node that is close to the sink. The energy consumption of the entire network is, therefore, greatly reduced, the network transmission rate is accelerated, and the network lifetime is improved.

**Figure 17:**
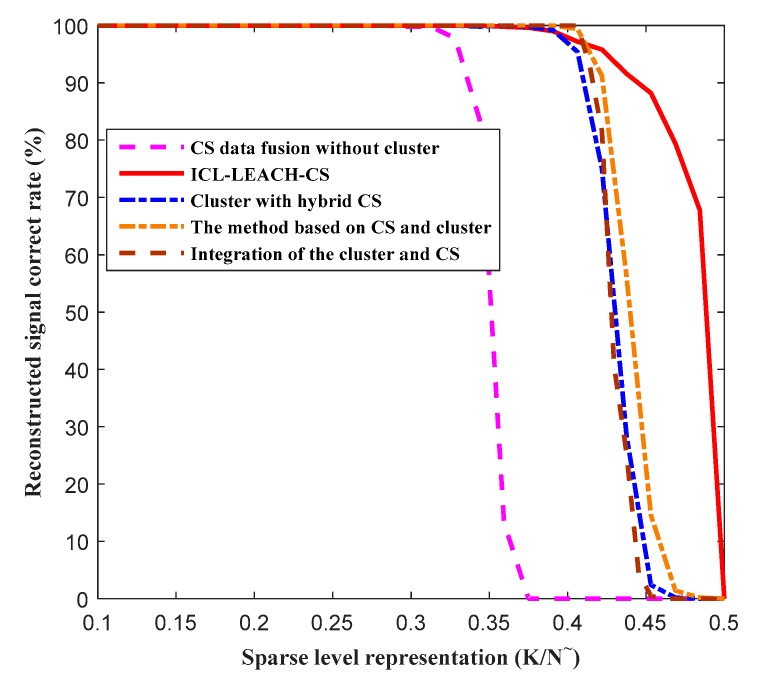
Reconstructed signal correct rate vs. the sparse level representation.

In addition, to improve the accuracy and credibility of data fusion in order to get a more accurate representation or estimation of the target, we need to analyze the correct rate of signal reconstruction. We compare the ICL-LEACH-CS algorithm with four other CS-based data fusion algorithms (i.e., the method based on CS and cluster, integration of the cluster and CS, cluster with hybrid CS, and CS data fusion without cluster). Figure 17 reveals the signal reconstruction accuracy as a function of the sparseness level (K/N), where K is the sparsity, and N is the signal length. When the sparse level is 0.31, the reconstruction accuracy of the five algorithms (i.e., the method based on CS and cluster, integration of the cluster and CS, ICL-LEACH-CS, cluster with hybrid CS, and CS data fusion without cluster) is 100%. It can be seen that the CS data fusion without cluster algorithm fails when the sparse level is 0.31, but the other four algorithms (the method based on CS and cluster, integration of the cluster and CS, ICL-LEACH-CS, and cluster with hybrid CS) still function. When the sparsity reached 0.385, the Cluster with hybrid CS algorithm also started to fail. However, ICL-LEACH-CS continues until K/N=0.5, which is almost equal to half of the measured amount. This also shows that the proposed algorithm’s reconstruction accuracy is relatively high.

## 5. Conclusions

In this paper, we studied a data fusion scheme for WSNs. Unlike the traditional clustering data fusion strategy, we considered a combination of clustering and compressed sensing (CS). In this way, we investigated the data fusion mechanism based on the improved LEACH clustering algorithm and CS, which we named the improved clustered LEACH and CS (ICL-LEACH-CS) algorithm. As a new data processing method, CS can greatly reduce the amount of data generated by the sampling process. CS technology is used in the WSN clustering algorithm to compress the data monitored by the node and then transmit it into the network, which can greatly reduce the energy consumption. Since the LEACH algorithm has high randomness, we consider the distance, residual energy, and compression ratio and propose an improved LEACH algorithm called ICL-LEACH. The ICL-LEACH algorithm is used to select the optimal cluster head (CH) node. The cluster member (CM) node sends the data to the CH node. The CH reuses the CS to compress the data and finally sends the compressed data to the sink node. The sink node reconstructs the data and restores the original information. When sparse representation of the signal, we choose the classical Fourier transform (FFT) sparse method. The method has the advantages of good stability and low computational complexity. When designing the measurement matrix, we choose an independent and identically distributed Gaussian random matrix as the observation matrix. We also prove that the sparse basis and the observation base are irrelevant, and the RIP is guaranteed. When designing the signal recovery algorithm, we translate the optimization problem into an optimization problem under the $\ell_{1}$ minimum norm and use the sub-gradient iterative algorithm to get accurate results. In addition, we analyzed the time complexity of the ICL-LEACH-CS and also proved its convergence. The simulation results show that the ICL-LEACH-CS algorithm greatly reduces the energy consumption of a WSN node compared to the traditional clustering algorithm, prolongs the network’s lifetime, and balances the network load of the node. These results give further insight into ways to process data fusion in WSNs in the future.

## Figures and Tables

**Table 1:** Simulation parameters.

Parameter	Value	Parameter	Value
Node number N	6/100	Noise N_*m*_	0.1
Transmission range	50 m	Sparsity K	25
Initial energy $E_{\mathrm{initial}}$	3 J	Measurement $\tilde{M}$	50
Data size l	4000 bit	Carrier frequency f	25 kHz
$E_{elec}$	50 nJ/bit	Control packet length N	100 bit
$E_{amp}$	100 pJ/(bit·m^2^)	Sampling frequency fs	100 kHz
$E_{fs}$	10 pJ/(bit·m^2^)	Setup phase: stabilization phase	1:15
*E* *_df_*	5 nJ/bit	Maximum round R	5000
CH selection ratio p	0.05	ξ	$1\times e^{-9}$

## Appendix A

**Definition 1.** 
*For the* K *sparse signal* X *, where* K *is a isnormal and* K=1,2⋯ *, if the minimum value of the isometry constant* $\delta_{K}\in \left(0,1\right)$ *satisfies Equation (A1)*

(A1) $$\left(1-\delta_{K}\right){\left\|X\right\|}_{2}^{2}\leq \left\|\Phi_{T}X\right\|\leq \left(1+\delta_{K}\right){\left\|X\right\|}_{2}^{2}$$

*then the* $\tilde{M}\times \tilde{N}$ *matrix* Φ *satisfies* K *order RIP [6].*

The RIP of order 2K implies that for any K sparse signal X, if the measurement matrix Φ satisfies the 2K order RIP

(A2) $$\left(1-\delta_{2K}\right){\left\|X\right\|}_{2}^{2}\leq \left\|\Phi_{T}X\right\|\leq \left(1+\delta_{2K}\right){\left\|X\right\|}_{2}^{2}$$

then Y=ΦX has a unique solution.

The RIP ensures that the measurement matrix does not map two different sparse signals into the same set (i.e., it can guarantee one-to-one mapping between the original space and the sparse space).

## Appendix B

As discussed before, the measurement vector is denoted as $y_{m}=\phi_{m}x_{m}+\sigma_{m}$, where $x_{m}\in X_{m}$, $\phi_{m}\in \Phi_{m}\in {\mathbb{R}}^{\tilde{M}\times \tilde{N}}$, and $\sigma_{m}$ are the Gaussian noise. Let ${\tilde{x}}_{m}^{*}$ denote the reconstruction value of the t+1 update, and the expression is as follows:

(A3) $$x_{m}=\left(y_{m}-\sigma_{m}\right)\phi_{m}^{T}$$

(A4) $$x_{m}^{t}=\left(y_{m}^{t}-\sigma_{m}^{t}\right){\left(\phi_{m}^{t}\right)}^{T}$$

Additionally, the error between the reconstructed signal and the original signal is $\Delta x=\left|{\tilde{x}}_{m}^{*}-x_{m}\right|$, as shown below:

(A5) $$\begin{array}{ll} {\tilde{x}}_{m}^{*} & =x_{m}^{t+1}\\ & =x_{m}^{t}+{\Delta x}^{t}\\ & =\left(y_{m}^{t}-\sigma_{m}^{t}\right){\left(\phi_{m}^{t}\right)}^{T}+\Delta x^{t} \end{array}$$

where ${\tilde{x}}_{m}^{*}$ represents the value before the update and $\Delta x^{t}$ is the error of the *t*-th update.

(A6) $$\begin{array}{ll} \Delta x & =\left|{\tilde{x}}_{m}^{t}{}^{*}-x_{m}^{t}\right|\\ & =\left|\left(\left(y_{m}^{t}-\sigma_{m}^{t}\right)\phi_{m}^{T}+\Delta x^{t}\right)-\left(\left(y_{m}^{t}-\sigma_{m}^{t}\right){\left(\phi_{m}^{t}\right)}^{T}\right)\right|\\ & =\left|\Delta x^{t}\right| \end{array}$$

As the number of iterations t increases, the error between the optimal solution obtained by the convex optimization and the original signal becomes smaller and smaller. When t→∞, $\Delta x^{t}\to 0$, so $x_{m}$ converges to ${\tilde{x}}_{m}^{*}$.
